# The N-Terminal Domain of Human DNA Helicase Rtel1 Contains a Redox Active Iron-Sulfur Cluster

**DOI:** 10.1155/2014/285791

**Published:** 2014-07-24

**Authors:** Aaron P. Landry, Huangen Ding

**Affiliations:** Department of Biological Sciences, Louisiana State University, 202 Life Sciences Building, Baton Rouge, LA 70803, USA

## Abstract

Human telomere length regulator Rtel1 is a superfamily II DNA helicase and is essential for maintaining proper length of telomeres in chromosomes. Here we report that the N-terminal domain of human Rtel1 (RtelN) expressed in *Escherichia coli* cells produces a protein that contains a redox active iron-sulfur cluster with the redox midpoint potential of −248 ± 10 mV (pH 8.0). The iron-sulfur cluster in RtelN is sensitive to hydrogen peroxide and nitric oxide, indicating that reactive oxygen/nitrogen species may modulate the DNA helicase activity of Rtel1 via modification of its iron-sulfur cluster. Purified RtelN retains a weak binding affinity for the single-stranded (ss) and double-stranded (ds) DNA *in vitro*. However, modification of the iron-sulfur cluster by hydrogen peroxide or nitric oxide does not significantly affect the DNA binding activity of RtelN, suggesting that the iron-sulfur cluster is not directly involved in the DNA interaction in the N-terminal domain of Rtel1.

## 1. Introduction

In vertebrates, telomeres are the protective structures at the end of chromosomes and are composed of a repetitive TTAGGG sequence and associated proteins that form a core structure known as the Shelterin complex [1, 2]. Telomeres become shorter with each round of DNA replication and are compensated for by telomerase [3]. In addition to telomerase, the telomere length is regulated by other genetic [4] and epigenetic [5] factors. Among them, telomere length regulator 1 (Rtel1) has an essential role in maintaining proper length of telomeres [4, 6]. Deletion of Rtel1 in mice is embryonic lethal with increased incidence of chromosomal abnormalities and telomere loss [4]. In humans, Rtel1 is highly expressed in several types of tumor tissues [7] and specific mutations in Rtel1 have been attributed to dyskeratosis congenita and Hoyeraal-Hreidarsson syndrome [8, 9].

Human Rtel1 is a superfamily II DNA helicase [10] and is homologous to other human DNA helicases XPD (Xeroderma pigmentosum factor D) [11], FancJ (Fanconi's anaemia complementation group J)/BACH1 (for BRCA1-associated C-terminal helicase) [12, 13], and ChlR1 (a protein required for normal mitotic progression) [14]. However, unlike other DNA helicases, Rtel1 preferentially disrupts the D-loop of the T-loop structure formed at the end of telomeres [15] and may act as an antirecombinase to prevent formation of D-loop [6, 16]. In the absence of Rtel1, the T-loop structure could be erroneously resolved as a substrate for homologous recombination [16], leading to telomere deficiency. On the other hand, excessive activity of Rtel1 would be detrimental as increased Rtel1 helicase activity would disengage the T-loop structure, leading to telomere deprotection and genomic instability [16]. Thus, the helicase activity of Rtel1 must be tightly regulated to maintain proper length of telomeres in chromosomes [6].

**Figure 1:**
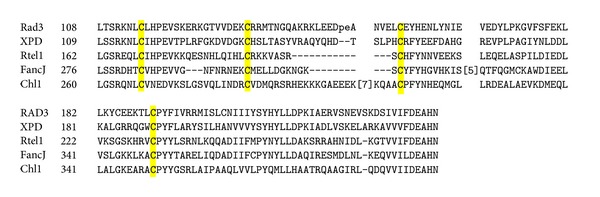
Alignment of the N-terminal domain of some human DNA helicases. Sequence alignment of yeast DNA helicase Rad3 and human DNA helicase XPD, Rtel1, FancJ, and Chl1 was generated by cobalt (NCBI). Four conserved cysteine residues proposed for hosting an iron-sulfur cluster in these DNA helicases are highlighted in yellow.

The sequence alignment analyses revealed that the N-terminal domain of human Rtel1 contains a conserved region for hosting a putative iron-sulfur cluster via four cysteine residues (Figure 1) [17]. It has previously been reported that the DNA helicase Rad3 from yeast [18], XPD homologues from archaea [19–22], DNA-damage-inducible DNA helicase DinG from* Escherichia coli* [23], and AddAB-type helicase-nuclease from* Bacillus subtilis* [24] contain a [4Fe-4S] cluster essential for the helicase activity. However, the existence of the iron-sulfur cluster in any human DNA helicases has not been experimentally demonstrated. Here, we report that expression of the N-terminal domain (residues 1–312) of human Rtel1 (RtelN) in* E. coli* cells produces a protein that contains a redox active iron-sulfur cluster with redox midpoint potential (*E*_*m*7_) of −248 ± 10 mV (pH 8.0). Purified RtelN retains a weak binding activity for the single-stranded (ss) and double-stranded (ds) DNA, and disruption of the iron-sulfur cluster by hydrogen peroxide or nitric oxide does not affect the DNA binding activity of RtelN, suggesting that iron-sulfur cluster in the N-terminal domain may not be directly involved in the DNA interaction in Rtel1.

## 2. Materials and Methods

### 2.1. Protein Preparation

The DNA fragment encoding the N-terminal domain (residues 1–312) (RtelN) of human regulator of telomere length 1 (Rtel1) was synthesized for expression in* E. coli* cells (Genescript co.). The gene was subcloned into an expression plasmid pET28b^+^ which was introduced into* E. coli* BL21 cells. The* E. coli* cells hosting the expression plasmid were grown in LB media to an OD_600 nm_ of ~0.6 before isopropyl *β*-D-1-thiogalactopyranoside (200 *μ*M) was added to induce the protein expression for three hours. The cells were harvested and passed through French press once. Recombinant RtelN in pellets was solubilized by adding urea (6 M), and the protein was purified using a nickel-agarose column attached to a FPLC system (GE Biosciences), followed by passing through a HiTrap desalting column. The molecular weight of RtelN was confirmed by the MALDI mass spectrometer (Chemistry Department, LSU). The concentration of purified RtelN was measured from the absorption peak at 280 nm using an extinction coefficient of 25.5 mM^−1^ cm^−1^. The total iron content in purified RtelN sample was determined using an iron indicator FerroZine [25]. The total acid-labile sulfide content was determined according to Siegel's method [26]. The single-stranded DNA binding protein SSB [27] was prepared as described previously [28].

### 2.2. The DNA Binding Activity Assay of RtelN

The DNA binding activity assay was carried out using a fluorescence labeled 40 mer (5′-F*-AATTGCGATCTAGCTCGCCAGUAGCGACCTT ATCTGATGA-3′). For single-stranded (ss) DNA binding assay, the 40 mer (0.5 *μ*M) was incubated with increasing concentrations of protein in buffer containing Tris (20 mM, pH 8.0), NaCl (50 mM), *β*-mercaptoethanol (1 mM), MgCl_2_ (1 mM), and bovine serum albumin (0.5 mg/mL). For double-strand (ds) DNA binding assay, the fluorescence labeled 40 mer was annealed to a complementary ssDNA in an annealing buffer containing Tris (50 mM, pH 8.0), NaCl (50 mM), and MgCl_2_ (10 mM). Prepared dsDNA labeled with fluorescence was incubated with increasing concentrations of protein in buffer as described above. After incubation at room temperature for 15 min, samples were loaded on to a 0.6% agarose gel in TAE buffer. The agarose gel was run at 10 V per cm for 30 min at room temperature and photographed in a KODAK Gel Logic 200 Imaging System.

### 2.3. Redox Titration of the RtelN Iron-Sulfur Cluster

A specially-designed cuvette was used for redox titration experiments as described by Leslie Dutton [29]. Briefly, purified RtelN (20 *μ*M) dissolved in buffer containing Tris (50 mM, pH 8.0) and NaCl (500 mM) was incubated with a redox mediator safranin O (1 *μ*M) in a sealed cuvette and equilibrated with pure argon gas for 45 minutes at room temperature. The redox potential was adjusted by adding a small amount of freshly prepared sodium dithionite using a gas-tight 10-*μ*L Hamilton micro-syringe (Hamilton Co., Reno, NV). The redox potential was monitored with a redox microelectrode (Microelectrodes Inc., Bedford, NH) which was calibrated using a standard ZoBell solution (*E*_*h*_ = +238 mV) containing potassium ferricyanide (5 mM) and potassium ferrocyanide (5 mM) in buffer containing Tris (20 mM, pH 8.0) and NaCl (500 mM). The redox titration data were fitted to the Nernst equation with *n* = 1 using KaleidaGraph (Synergy Software co.).

### 2.4. Hydrogen Peroxide and Nitric Oxide Treatments of RtelN

For hydrogen peroxide (H_2_O_2_) treatments, purified RtelN was incubated with different concentrations of H_2_O_2_ at room temperature for 30 min, followed by repurification of the protein from the incubation solutions. For nitric oxide (NO) treatments, purified RtelN dissolved in a sealed vial was purged with pure argon gas for 15 min, followed by incubation with the NO-releasing reagent diethylamine NONOate (Cayman Chemicals co.) at 37°C for 10 min. RtelN was repurified after the NO treatment. Modification of the iron-sulfur cluster in RtelN by H_2_O_2_ or NO was quantified by the UV-visible absorption spectrometer.

### 2.5. The Circular Dichroism (CD) and Electron Paramagnetic Resonance (EPR) Measurements

The circular dichroism (CD) spectra were recorded on a Jasco J-815 CD spectrometer (AgCenter Biotechnology Laboratories, LSU) at room temperature. The composition of secondary structures was obtained using the CDNN program [30]. The electron paramagnetic resonance (EPR) spectra were recorded at X-band on a Bruker ESR-300 spectrometer equipped with an Oxford Instruments 910 continuous flow cryostat. EPR conditions were as follows: microwave frequency, 9.45 GHz; microwave power, 10 mW; modulation frequency, 100 kHz; modulation amplitude, 2 mT; sample temperature, 10 K; receive gain, 1 × 10^5^.

## 3. Results

### 3.1. The N-Terminal Domain of Human Rtel1 Hosts an Iron-Sulfur Cluster

**Figure 2:**
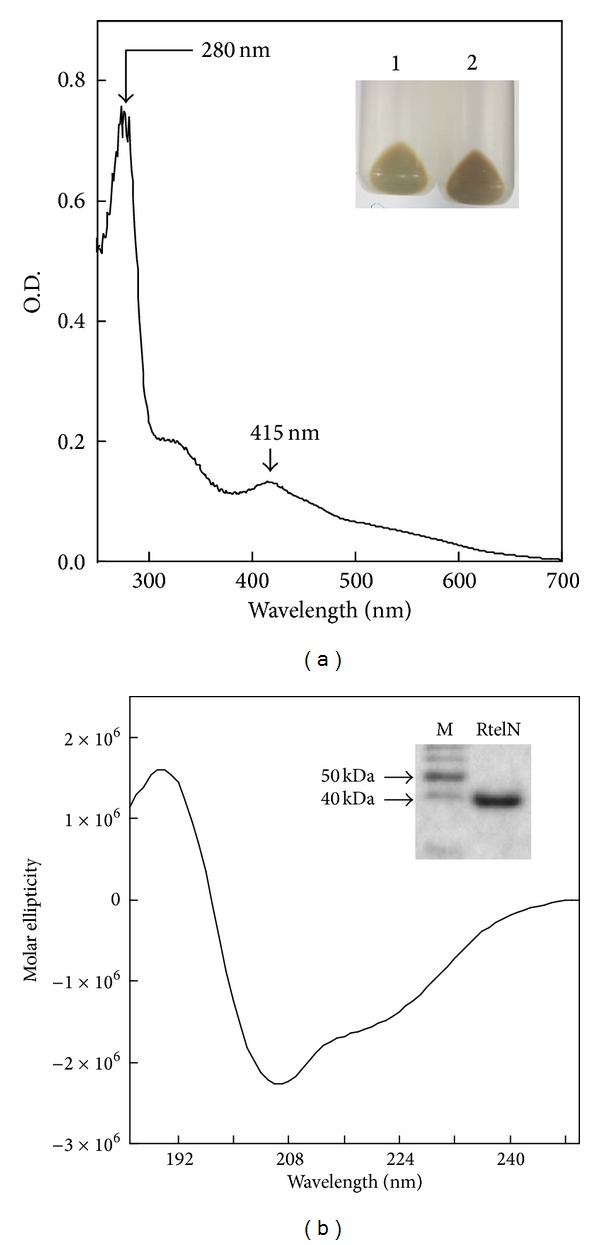
N-terminal domain of Rtel1 (RtelN) contains an iron-sulfur cluster. (a) UV-visible absorption spectrum of purified RtelN. Purified RtelN (30 *μ*M) was dissolved in buffer containing Tris (20 mM, pH 8.0) and NaCl (500 mM). The absorption peak at 415 nm indicates an iron-sulfur cluster in RtelN. Inset is a photograph of cell pellets after induction with (sample 2) or without (sample 1) IPTG (200 *μ*M). (b) Circular dichroism (CD) spectrum of purified RtelN. Purified RtelN (6.75 *μ*M) was dissolved in potassium phosphate buffer (10 mM, pH 8.0). The spectrum was an average of three scans. Insert is a photograph of the SDS-PAGE gel of purified RtelN. Left lane, molecular marker (M); right lane, purified RtelN.

When the N-terminal domain of human Rtel1 (RtelN) was expressed in* E. coli* cells, the cell pellets had a dark-red color (Figure 2(a) insert). Recombinant RtelN was purified from the* E. coli* cells as described in the Materials and Methods. The UV-visible absorption measurements showed that purified RtelN had an absorption peak at 415 nm (Figure 2(a)), similar to that of* S. acidocaldarius* XPD [4Fe-4S] cluster [22] and* E. coli* DinG [4Fe-4S] cluster [23]. Purified RtelN was further subjected to the Circular dichroism (CD) measurements. As shown in Figure 2(b), purified RtelN adopted an ordered structure with about 25% alpha-helix, 32% beta-sheet, 20% beta turns, and 22% random coil. The iron and sulfide content analyses revealed that purified RtelN contained 0.83 ± 0.13 iron and 0.75 ± 0.16 acid-labile sulfide per protein.

The low iron and sulfide contents in purified RtelN could be due to the protein purification process under denaturation conditions. To fully reconstitute the iron-sulfur clusters in RtelN, renatured protein was reconstituted with excess iron and sulfide as described previously [23]. After reconstitution, the iron and sulfide contents in RtelN were increased to 3.5 ± 0.3 iron and 3.2 ± 0.5 sulfide per RtelN, respectively, indicating that each RtelN monomer may bind a [4Fe-4S] cluster.

### 3.2. The Iron-Sulfur Cluster in RtelN Is Redox Active

**Figure 3:**
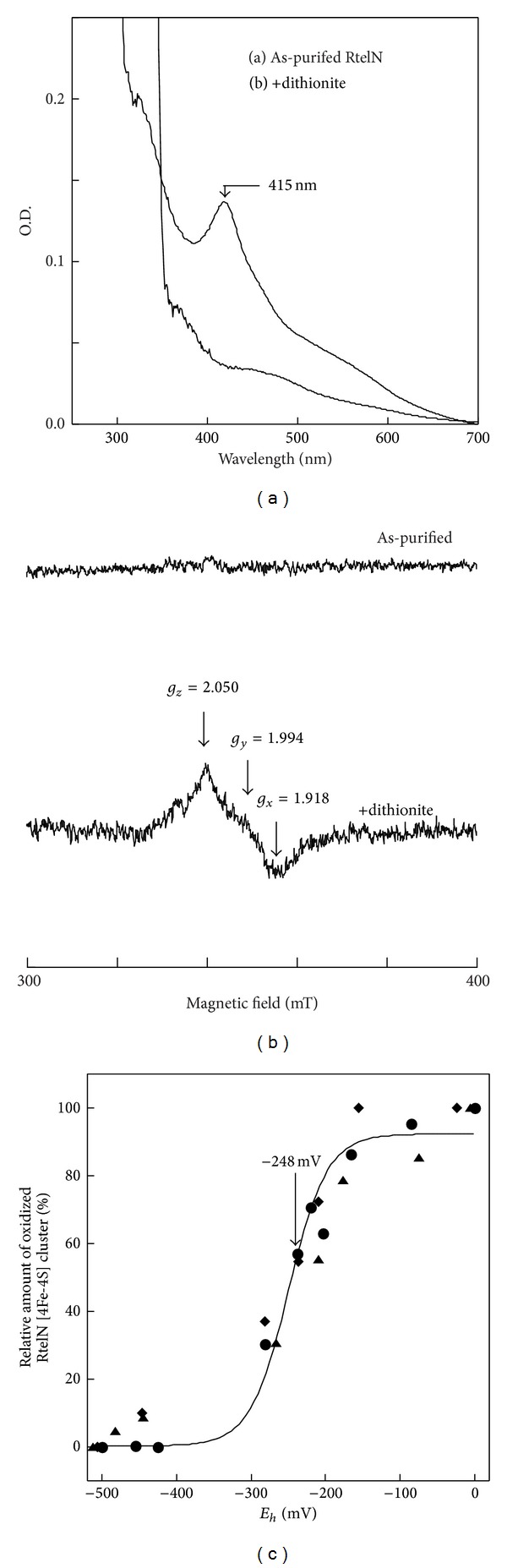
Redox titration of the RtelN iron-sulfur cluster. (a) UV-visible spectra of purified RtelN. Purified RtelN (40 *μ*M) (spectrum 1) was reduced with freshly prepared sodium dithionite (2 mM) (spectrum 2). (b) EPR spectra of purified RtelN. RtelN (90 *μ*M) (spectrum 1) was reduced with freshly prepared sodium dithionite (2 mM) (spectrum 2). (c) Redox titration of purified RtelN. The amplitudes of the absorbance peak at 415 nm were normalized to 0 and 100% for the fully reduced and oxidized RtelN iron-sulfur cluster in solution, respectively. The solid line drawn through three sets of data points represents the best fit to a Nernst equation (*n* = 1) with *E*_*m*_ = −248 ± 10 mV.

When freshly prepared sodium dithionite was added to the solution containing RtelN, the absorption peak at 415 nm of the RtelN iron-sulfur cluster was completely eliminated (Figure 3(a)). The absorption peak at 415 nm was restored when the reduced RtelN iron-sulfur cluster was reoxidized by oxygen (data not shown), suggesting that the RtelN iron-sulfur cluster can be reversibly reduced. This notion was further confirmed by the electron paramagnetic resonance (EPR) measurements: while purified RtelN was EPR silent, addition of sodium dithionite to purified RtelN produced an EPR spectrum with *g*_*x*_ = 1.918, *g*_*y*_ = 1.994, and *g*_*z*_ = 2.050 (Figure 3(b)), a spectrum similar to that of the reduced* E. coli* DNA helicase DinG [4Fe-4S] cluster [23].

Redox titration experiments were carried out to determine the redox midpoint potential (*E*_*m*_) of the RtelN iron-sulfur cluster. The amplitude of the absorption peak at 415 nm of RtelN was plotted as a function of redox potentials in the solution (Figure 3(c)). The data from three sets of experiments were fitted to a Nernst equation (*n* = 1) with an *E*_*m*8_ of −248 ± 10 mV, which is about 140 mV higher than that of the* E. coli* DinG [4Fe-4S] cluster [23].

### 3.3. Purified RtelN Has a Weak DNA Binding Activity

**Figure 4:**
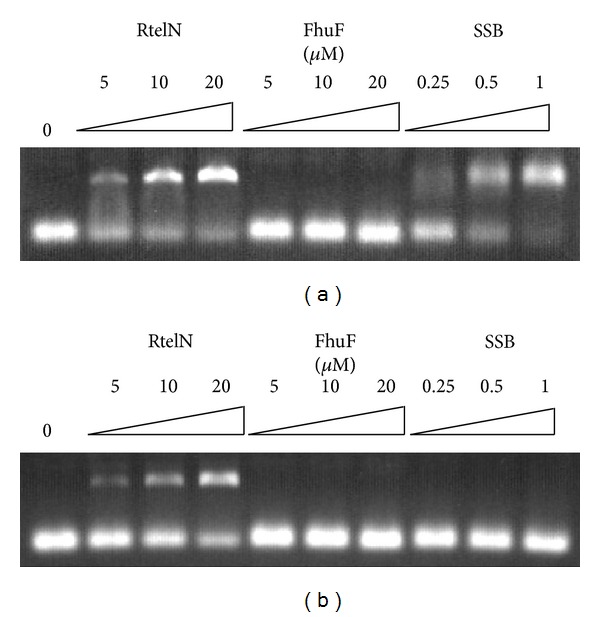
DNA binding activity of purified RtelN. (a) ssDNA binding activity of RtelN. A fluorescence-labeled ssDNA (0.5 *μ*M) was incubated with the indicated amount of protein. FhuF is an iron-sulfur protein that has no DNA binding activity. SSB is* E. coli* ssDNA binding protein. The DNA-protein complex and free DNA probe were resolved on a 0.6% agarose gel. (b) dsDNA binding activity of RtelN. A fluorescence-labeled dsDNA (0.5 *μ*M) was incubated with the indicated amount of protein. The DNA-protein complex and free DNA probe were resolved on a 0.6% agarose gel. The results are representatives of three independent experiments.

The N-terminal domain of the archaeal DNA helicase XPD comprises part of the catalytic center [20]. To test whether the N-terminal domain of Rtel1 also contributes to the catalytic site, we examined the DNA binding activity of purified RtelN. As shown in Figure 4(a), purified RtelN formed a protein-DNA complex with the single-stranded (ss) DNA. FhuF, an iron-sulfur protein with a similar molecular weight as RtelN but with no known DNA binding activity [31], failed to bind any ssDNA, indicating that the ssDNA binding in RtelN is specific. Nevertheless, compared with the single-stranded DNA binding protein SSB [27], the binding affinity of RtelN for ssDNA was at least 10-fold-weaker. In parallel, we also determined the double-stranded (ds) DNA binding activity of RtelN under the same experimental conditions.  Figure 4(b) shows that RtelN could also bind dsDNA with the similar binding affinity as for ssDNA. In contrast, both FhuF and SSB did not bind any dsDNA as expected. Thus, purified RtelN has a binding activity for both ssDNA and dsDNA* in vitro*.

### 3.4. The Iron-Sulfur Cluster Is Not Required for the DNA Binding Activity of RtelN

**Figure 5:**
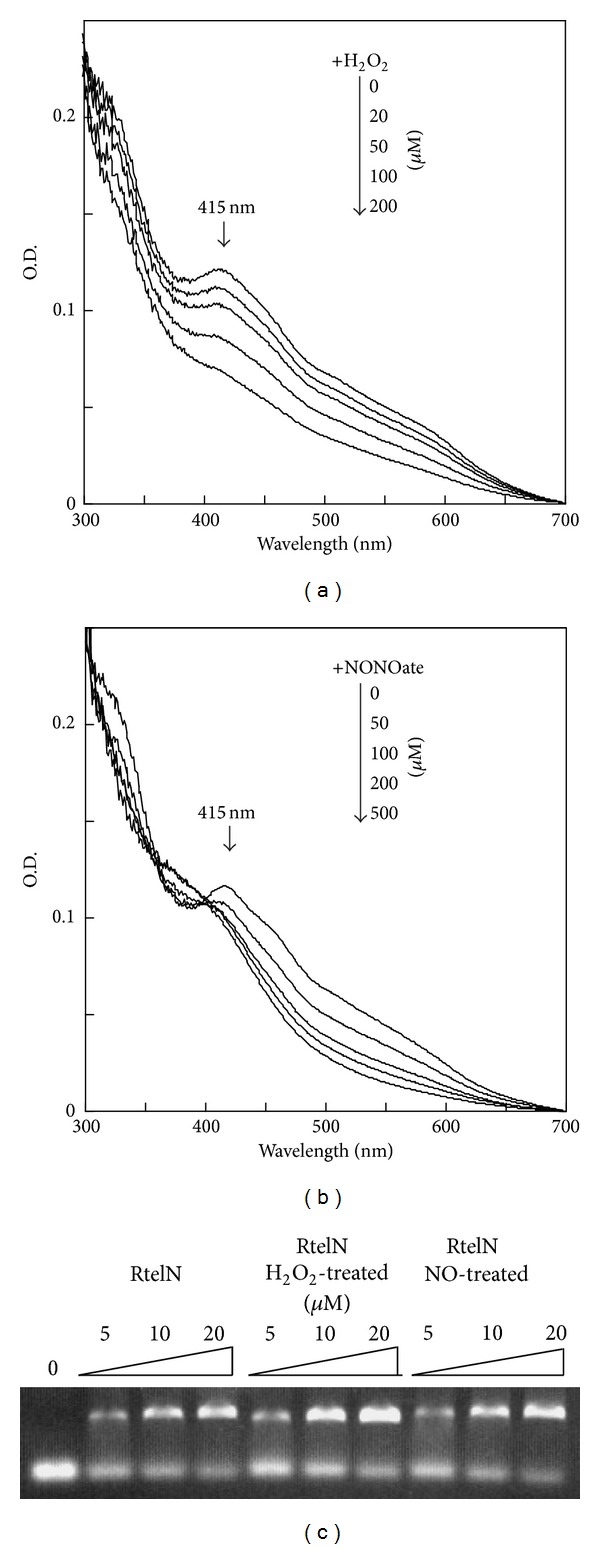
DNA binding activity of RtelN with modified iron-sulfur cluster. (a) Effect of H_2_O_2_ on the RtelN iron-sulfur cluster. RtelN (20 *μ*M) was incubated with the indicated concentrations of H_2_O_2_ (0 to 200 *μ*M) at room temperature for 30 min. The UV-visible spectra were taken after incubation. (b) Effect of NO on the RtelN iron-sulfur cluster. RtelN (20 *μ*M) was incubated with the indicated concentrations of the NO releasing reagent diethylamine NONOate (0 to 500 *μ*M) at 37°C for 10 min. The UV-visible spectra were taken after incubation. (c) ssDNA binding activity of RtelN after the iron-sulfur cluster was modified. Untreated RtelN and RtelN treated with 200 *μ*M H_2_O_2_ or 500 *μ*M NONOate were incubated with the fluorescence-labeled ssDNA (0.5 *μ*M). The DNA-protein complex and free DNA probe were resolved on a 0.6% agarose gel. The results are representative of three independent experiments.

Ironically, iron-sulfur clusters in proteins are often sensitive to reactive oxygen species [32] and nitrogen species [33, 34]. To test if the iron-sulfur clusters in RtelN can be modified by reactive oxygen species, we incubated RtelN with hydrogen peroxide at room temperature.  Figure 5(a) shows that addition of increasing amounts of hydrogen peroxide removed the absorption peak at 415 nm of purified RtelN, indicating that the iron-sulfur cluster in RtelN are disrupted by hydrogen peroxide. Purified RtelN was also incubated with the nitric oxide-releasing reagent NONOate in solution. Again, the absorption peak at 415 nm of the RtelN iron-sulfur cluster was largely abolished as the concentration of nitric oxide was increased (Figure 5(b)). Thus, the iron-sulfur cluster in RtelN is sensitive to both hydrogen peroxide and nitric oxide.

We then examined the DNA binding activity of RtelN after the protein was treated with hydrogen peroxide and nitric oxide.  Figure 5(c) shows that the DNA binding activity of RtelN remained almost the same when the iron-sulfur cluster was modified by hydrogen peroxide or nitric oxide, suggesting that iron-sulfur cluster is not required for the DNA binding activity of RtelN.

## 4. Discussion

Recent studies have identified a new set of iron-sulfur cluster-containing enzymes that are involved in DNA processing in bacteria and eukaryotic cells [17]. Among these enzymes are a group of DNA helicases that require an intact iron-sulfur cluster for the DNA helicase activity [11, 35]. For example, it has been shown that the DNA helicase Rad3 from yeast and XPD homologues from archaea contain a [4Fe-4S] cluster essential for the enzyme activity [19–22]. In human XPD, mutations in the N-terminal domain that hosts a putative iron-sulfur cluster have been associated with several genetic diseases including xeroderma pigmentosum [20]. Interestingly, in addition to XPD, humans have at least three other DNA helicases: FancJ (Fanconi's anaemia complementation group J)/BACH1 (for BRCA1-associated C-terminal helicase) [12, 13], ChlR1 (a protein required for normal mitotic progression) [14], and Rtel1 of telomere length regulation [4, 6] that contain a putative iron-sulfur cluster binding site in the N-terminal domain (Figure 1). However, the existence of the [4Fe-4S] clusters in any of these human DNA helicases has not been experimentally demonstrated. Here we find that the N-terminal domain of human Rtel1 (RtelN) expressed in* E. coli* cells contains a redox active [4Fe-4S] cluster and that the iron-sulfur cluster in purified RtelN is highly sensitive to hydrogen peroxide and nitric oxide. The results suggest that human Rtel1, like XPD from archaea [19–22], likely contains a [4Fe-4S] cluster.

Despite the findings of the iron-sulfur clusters in these DNA helicases, specific function of the [4Fe-4S] clusters in the DNA helicases remains largely elusive [17]. In previous studies, we reported that oxidation of the reduced iron-sulfur cluster in* E. coli* DNA helicase DinG reversibly switches on the enzyme activity, and proposed that iron-sulfur cluster may regulate the helicase activity in response to redox signals [23]. Here, we have tested the idea further in the human DNA helicase Rtel1. While attempts to purify a full-length human Rtel1 from* E. coli* cells were not successful, we were able to prepare the soluble N-terminal domain (residues 1–312) of human Rtel1 (RtelN). The results demonstrated that RtelN contains a redox active iron-sulfur cluster with a redox midpoint potential of −248 ± 10 mV (pH 8.0) (Figure 3). The redox potential in cytosol and nucleus of mammalian cells has been reported to be around −325 mV (pH 7.0) [36]. However, when cells are under oxidative stress or during apoptosis and differentiation, the intracellular redox potential could increase to as high as +200 mV [37]. Assuming the redox midpoint potential of the iron-sulfur cluster in Rtel1 is similar to that in RtelN, we would expect that the iron-sulfur cluster in Rtel1 is in reduced state in cells under normal physiological conditions. Under oxidative stress or during apoptosis and differentiation [37], the iron-sulfur cluster in Rtel1 would be fully oxidized. We envision that oxidation of the reduced iron-sulfur cluster in Rtel1, like that in the* E. coli* DinG [23], may change the DNA helicase activity of the protein in response to redox signals. Thus, fluctuation of intracellular redox potential may result in change of the Rtel1's DNA helicase activity by changing the redox state of the iron-sulfur cluster. While the iron-sulfur cluster appears to be dispensable for the DNA binding activity of purified RtelN (Figure 5), the iron-sulfur cluster could have an important role in other steps of the reaction catalyzed by Rtel1.

The telomere length of chromosomes has been linked to intracellular oxidative stress in human cells [38]. The finding that the RtelN iron-sulfur cluster is sensitive to hydrogen peroxide and nitric oxide (Figure 5) may provide a rational explanation for the association between the telomere length of chromosomes and oxidative stress in cells. Since the iron-sulfur cluster is essential for the DNA helicase activity of XPD from yeast and archaea [19–22], disruption of iron-sulfur clusters in protein would likely change the helicase activity of Rtel1 in human cells. If the iron-sulfur cluster in Rtel1 is as sensitive to hydrogen peroxide or nitric oxide as that in RtelN, the DNA helicase activity of Rtel1 could be modulated by intracellular reactive oxygen/nitrogen species. Therefore, modification of the iron-sulfur cluster in Rtel1 by reactive oxygen/nitrogen species could at least in part contribute to the telomere length of chromosomes in cells [38]. Evidently, additional experiments are required to illustrate the regulatory role of the iron-sulfur cluster in the human Rtel1 and other DNA helicases.

## Abbreviations

EPR: Electron paramagnetic resonance
*E*_*m*_: Midpoint redox potential
Rtel1: Human telomere length regulator
RtelN: The N-terminal domain of Rtel1
ssDNA: Single-stranded DNA
dsDNA: Double-stranded DNA.
